# The infection of turkeys and chickens by reassortants derived from pandemic H1N1 2009 and avian H9N2 influenza viruses

**DOI:** 10.1038/srep10130

**Published:** 2015-06-01

**Authors:** Honglei Sun, Weili Kong, Litao Liu, Yi Qu, Chong Li, Ye Shen, Yu Zhou, Yu Wang, Sizhe Wu, Juan Pu, Jinhua Liu, Yipeng Sun

**Affiliations:** 1 Key Laboratory of Animal Epidemiology and Zoonosis, Ministry of Agriculture, College of Veterinary Medicine, and State Key Laboratory of Agrobiotechnology, China Agricultural University, Beijing, China

## Abstract

Outbreaks of pandemic H1N1 2009 (pH1N1) in turkeys have been reported in several countries. Co-infection of pH1N1 and avian H9N2 influenza viruses in turkeys provide the opportunity for their reassortment, and novel reassortant viruses might further be transmitted to other avian species. However, virulence and transmission of those reassortant viruses in poultry remain unclear. In the present study, we generated 16 single-gene reassortant influenza viruses including eight reassortants on the pH1N1 background by individual replacement with a corresponding gene segment from H9N2 and eight reassortants on the H9N2 background replaced individually with corresponding gene from pH1N1, and characterized reassortants viruses in turkeys and chickens. We found that the pH1N1 virus dramatically increased its infectivity and transmissibility in turkeys and chickens after introducing any gene (except for PB2) from H9N2 virus, and H9N2 virus acquired single gene (except for HA) of pH1N1 almost did not influence its replication and transmission in turkeys and chickens. Additionally, 13 reassortant viruses transmitted from turkeys to chickens. Our results indicate that turkeys and chickens are susceptible to pH1N1-H9N2 reassortant viruses, and mixing breeding of different avian species would facilitate the transmission of these reassortant viruses.

Two pandemics in history were caused by avian influenza viruses that underwent reassortment with human influenza viruses, including the 1957 H2N2 Asian pandemic and the 1968 H3N2 Hong Kong pandemic^1^. Therefore, surveying the virulence and pathogenic mechanism of novel human-avian influenza reassortant viruses is necessary in the prevention and control of a potential influenza pandemic. High genetic compatibility between circulating human and avian influenza viruses has been recognized by artificially producing reassortant viruses using reverse genetics, including human H3N2 and avian H5N1, pandemic H1N1 2009 (pH1N1) and H5N1, as well as pH1N1 and H9N2 influenza viruses^2^, ^3^, ^4^. Of note, some of these reassortants possessed increased pathogenicity than parental viruses and droplet transmissiblitity in mammalians, indicating that these reassortants pose a significant threat to public health^2^,^3^,^4^,^5^.

pH1N1 influenza viruses spread by human-to-human transmission across the globe at an unprecedented rate to cause the first pandemic of the 21st century^6^,^7^, and continues to circulate in humans as a recurrent seasonal influenza virus^8^,^9^,^10^. Although the pH1N1 influenza virus has limited infectivity for poultry^11^, the presence of avian-origin genes in the pH1N1 virus increases the potential for infection in poultry^6^. In fact, pH1N1 infections have occurred in turkey breeder flocks in Canada, Chile, the United Kingdom and the United States, and were most likely through human-to-poultry transmission^12^,^13^,^14^,^15^. Avian H9N2 influenza viruses circulate worldwide and are endemic in multiple terrestrial avian species in Asia^16^,^17^. Recent studies have shown that H9N2 viruses donated six internal genes to the newly emerged H7N9 and H10N8 influenza viruses that caused human infections in China^17^,^18^. Since 1966, H9N2 viruses of the North American lineage have been primarily isolated from turkeys^18^. Thus, co-infection with the pH1N1 and H9N2 influenza viruses in turkeys provides the opportunity for reassortment between these viruses. Furthermore, the novel reassortant viruses in turkeys may transmit to other poultry species, such as chicken, which is a major terrestrial host for a wide variety of influenza viruses. Additionally, pH1N1 and avian H9N2 influenza viruses also possess other co-infected hosts, including humans and pigs. If pH1N1-H9N2 reasssortants are infective for avian species, they could also transmit from other hosts to poultry such as transmission of pH1N1 from humans to turkeys. However, their infectivity in poultry is still unclear.

In the present study, we generated a series of single-gene reassortant viruses derived from pH1N1 and avian H9N2 influenza viruses and evaluated the viral growth and polymerase activity *in vitro* and replication and transmission of reassortant viruses in turkeys and chickens.

## Results

### Generation of reassortant viruses by reverse genetics

The reverse genetics systems of pH1N1 virus A/Beijing/16/2009 (BJ09) and H9N2 virus A/chicken/Hebei/LC/2008 (HB08) had been established previously^4^. Then, we generated a set of 16 single-gene reassortants (“7 + 1” reassortants) derived from BJ09 and HB08 viruses by reverse genetics. These reassortant viruses included eight reassortants in the background of HB08 replaced individually with a corresponding gene segment from BJ09 and eight reassortants in the background of BJ09 replaced individually with corresponding gene segment from HB08 (Table 1).

### Growth kinetics of reassortant viruses *in vitro*

**Figure 1:**
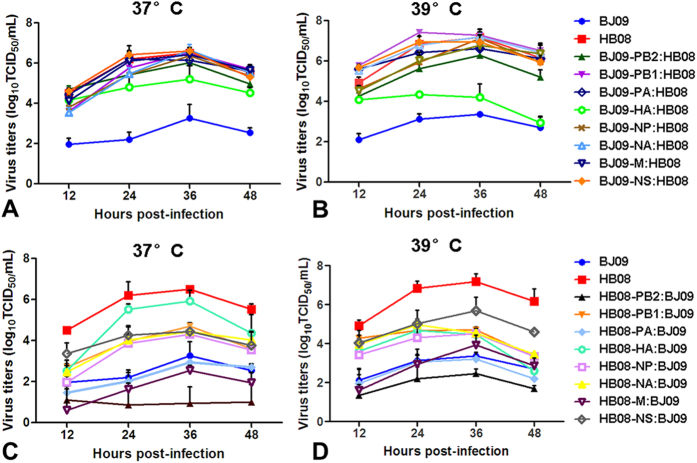
Viral growth kinetics of wild type and recombinant viruses in CEF cells. CEF cells were infected with BJ09, HB08 and their “7 + 1” reassortant viruses at an MOI of 0.001. Error bars above represent standard deviations from the means for three independent experiments.

The growth characteristics of the recombinant viruses and wild type viruses were determined in chicken embryonic fibroblast (CEF) cells at 37 °C or 39 °C which mimics the body temperature of chicken. CEF cells were infected with a multiplicity of infection (MOI) of 0.001 TCID_50_ per cell in the presence of 0.5 μg/mL TPCK trypsin. As shown in Fig. 1, the wild type HB08 avian influenza virus replicated efficiently in CEF cells with peak titers of 6.5 and 7.2 log_10_ TCID_50_/mL at 37 °C and 39 °C, respectively. In contrast, the BJ09 virus only reached its maximum titers of 3.3 and 3.4 log_10_ TCID_50_/mL at 37 °C and 39 °C, respectively. The peak titers in CEF cells at 37 °C or 39 °C of all the “7+1” reassortants in the backbone of HB08 were comparable to HB08 virus excepting for BJ09-PB2:HB08 and BJ09-HA:HB08 (*P *< 0.05) (Fig. 1A and 1B). For the “7+1” reassortants in the backbone of the BJ09 virus, despite the fact that all of them replicated inefficiently compared with the HB08 virus (*P *< 0.05), the replication of most reassortants significantly increased compared to the BJ09 virus at both 37 °C and 39 °C, except for HB08-PB2:BJ09, HB08-PA:BJ09 and HB08-M:BJ09 (*P *> 0.05) (Fig. 1C and 1D). We then evaluated the growth titer of the wild type and recombinant viruses in eggs at 72 h post-infection (hpi). The yield of wild type HB08 and BJ09 viruses in eggs were 9.3 ± 0 log_10_EID_50_/mL and 5.4 ± 0.1 log_10_EID_50_/mL, respectively (Table 1). The reassortants, with single BJ09 gene in the background of HB08, replicated efficiently in eggs with EID_50_ higher than 7.3 log_10_EID_50_/mL. The titers of the BJ09 virus increased by 0.3 to 3.3 logs after introducing individual genes from HB08, except for HB08-PB2:BJ09 with a titer of 4.8 ± 0.3 log_10_EID_50_/mL.

### Infection and transmission of reassortant viruses in turkeys

To understand the infectivity and transmissibility of pH1N1-H9N2 reassortant viruses in turkeys, we intranasally inoculated groups of ten six-week-old turkeys with 10^6^ EID_50_ of each virus in a 200-μL volume. After 24 h, the inoculated birds were placed in a new cage and housed with five naïve turkeys. Tracheal swabs were collected from all the birds at 3 and 5 days post-inoculation (dpi) and for virus titration. Sera were collected at 21 dpi for confirmation of seroconversion. The EID_50_ of HB08-PB2:BJ09 virus was too low; therefore, we did not include this virus in the animal experiment.

Isolation of BJ09 viruses was negative for all the birds, while seroconservation was observed in three of ten inoculated turkeys (Table 2). Wild type HB08 virus replicated efficiently in tracheas of all of the inoculated turkeys, and was transmitted to all the contact turkeys. After introducing single genes from BJ09 in the HB08 backbone, all of the tested reassortant viruses still replicated efficiently in the tracheas of inoculated turkeys, and were able to transmit to contact turkeys. Although the tested “7:1” reassortants in the BJ09 backbone did not replicate as well as those in HB08 backbone, all of them induced seroconversion of inoculated turkeys, and some viruses transmitted to the contact turkeys, including HB08-PB1:BJ09, HB08-PA:BJ09, HB08-NP:BJ09, HB08-NA:BJ09, and HB08-NS:BJ09. These results suggested that pH1N1 virus was able to infect and transmit in turkeys as long as they acquired single gene of avian origin.

### Turkey-to-chicken transmission of reassortant viruses

Our results indicate that turkeys could become the reservoir of pH1N1-H9N2 reassortant viruses. Different species of poultry were usually co-breeding in farmer household and live poultry markets. To investigate whether reassortant viruses could transmit from turkeys to other poultry such as chickens, we cohoused five naïve chickens with ten inoculated turkeys at 1 dpi for each virus group. Transmission was not observed between turkeys and chickens for wild type BJ09 pH1N1 virus, whereas wild type HB08 H9N2 virus transmitted to all the contact chickens with virus titer of 3.4 ± 0.3 log_10_EID_50_/mL at 4 days post-contact (dpc) (Table 1). Of the 15 tested pH1N1-H9N2 reassortant influenza viruses, 13 reassortant viruses induced contact chicken seroconversion indicating that they were able to transmit from turkeys to chickens, and 11 reassortant viruses were isolated in the trachea of contact chickens. Of note, BJ09-NA:HB08, BJ09-M:HB08 and BJ09-NS:HB08 viruses were shed from all of the contact chickens with viral titers higher than 10^3.5^ EID_50_/mL.

### Infection and transmission of reassortant viruses in chickens

Some pH1N1-H9N2 influenza reassortant viruses transmitted from turkeys to chickens. To understand the virulence of pH1N1-H9N2 reassortant viruses for chickens further, we performed an infection and transmission study in chickens. Groups of ten six-week-old SPF chickens were intranasally inoculated with 10^6^ EID_50_ of each virus. After 24 h, the inoculated birds were placed in a new cage and housed with five naïve chickens. Tracheal swabs were collected from all the chickens at 3 and 5 dpi for virus titration. At 3 dpi, three birds from each inoculated group were euthanized, and their lungs were collected and titrated by EID_50_ assay. Sera were collected at 21 dpi for confirmation of seroconversion.

BJ09 virus cannot infect chickens because neither virus production nor seroconversion was observed (Table 3). In contrast, the HB08 virus replicated efficiently in tracheas and lungs of all of the inoculated chickens and transmitted efficiently in chickens. Most of the pH1N1-H9N2 reassortant viruses replicated efficiently in the tracheas and lungs of inoculated chickens, especially BJ09-PB2:HB08, BJ09-PB1:HB08, BJ09-PA:HB08, BJ09-NA:HB08, BJ09-M:HB08, and BJ09-NS:HB08, the peak viral titers of which were higher than 10^4^ EID_50_/mL. We noticed that HB08-HA:BJ09 and HB08-M:BJ09 viruses, which could not transmit from turkeys to chickens, were able to infect chickens following inoculation of a high viral dose although they still could not transmit to naïve chickens. All the other tested reassortant viruses could transmit between chickens.

### *In vitro* viral polymerase activity of reassorted RNPs

**Figure 2:**
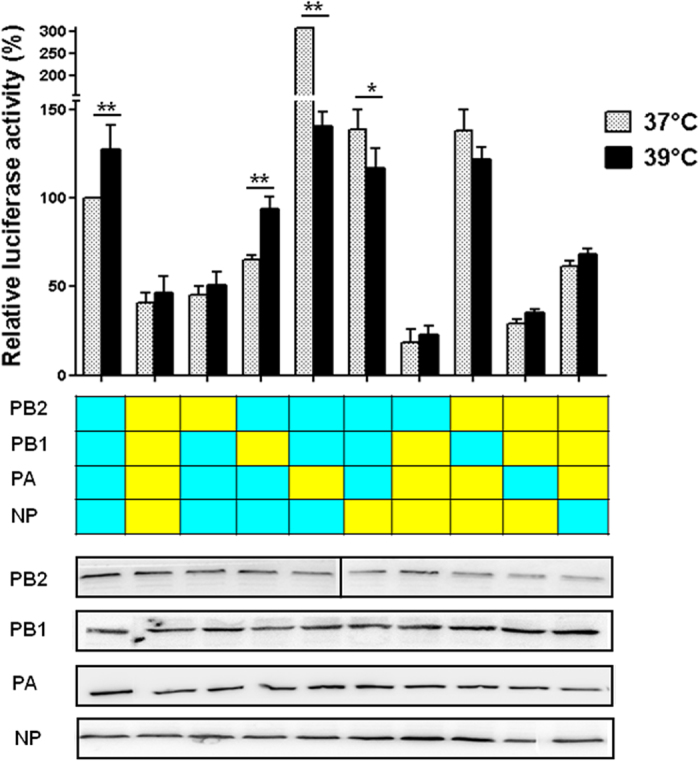
Polymerase activity of 8 RNP combinations between the BJ09 and HB08 viruses. The DF1 cells were transfected in duplicate with luciferase reporter plasmid p-Luci and internal control plasmid Renilla, together with plasmids expressing PB2, PB1, PA, and NP from either BJ09 or HB08 viruses. Segments derived from pH1N1 are in yellow, and those derived from HB08 virus are in blue. Cells were incubated at 37 °C (stippled bars) or at 39 °C (solid bars) for 24 h, and cell lysates were analyzed to measure Firefly and Renilla luciferase activities. Values shown are the mean ± SD of the three independent experiments and are standardized to those of HB08 measured at 37 °C (100%). * and ** indicate that the polymerase activity between 37 °C and 39 °C was significantly different with *P *< 0.05 and *P *< 0.01, respectively (ANOVA). PB2, PB1, PA, and NP expressions were detected by Western blot analysis.

Previous studies demonstrated that the viral ribonucleoprotein (RNP) complex had an important correlation with viral generation, replication, and pathogenicity^19^,^20^. To study the mechanisms underlying the differences in the phenotypes of the pH1N1-H9N2 reassortants in cells and poultry, we determined the activity of 16 RNP combinations of PB2, PB1, PA, and NP from either HB08 or BJ09 viruses by measuring the activity of luciferase at 37 °C or 39 °C in DF1 cells. The polymerase activity of BJ09 RNP (B_PB2_B_PB1_B_PA_B_NP,_ “B” stands for BJ09 virus) were approximately 60% and 80% lower than those of HB08 (H_PB2_H_PB1_H_PA_H_NP,_ “H” stands for HB08 virus) at 37 °C and 39 °C, respectively (Fig. 2). Introducing the PA gene from the BJ09 virus significantly increased the polymerase activity of HB08 RNP at 37 °C (*P *< 0.05), but not at 39 °C. In contrast, replacing the PA gene of HB08 by BJ09 reduced their RNP activity at both 37 °C and 39 °C (*P *< 0.05). Of note, the RNP activities of B_PB2_H_PB1_H_PA_H_NP_ and H_PB2_B_PB1_B_PA_B_NP_ were the lowest of all corresponding single-gene reasserting RNPs, indicating that the PB2 gene of BJ09 and HB08 was incompatible with RNP genes from other viruses in the DF1 cells. The low polymerase of H_PB2_B_PB1_B_PA_B_NP_ and B_PB2_H_PB1_H_PA_H_NP_ explained the inefficient replication of HB08-PB2:BJ09 and HB08-PA:BJ09 viruses in CEF cells and birds. The polymerase activity of H_PB2_H_PB1_H_PA_H_NP_, H_PB2_B_PB1_H_PA_H_NP_, H_PB2_H_PB1_B_PA_H_NP_ and H_PB2_H_PB1_H_PA_B_NP_ between 37 °C and 39 °C were significantly different (*P *< 0.05). Western blot analysis showed that the RNP proteins PB2, PB1, PA, and NP were expressed at similar levels, excluding the possibility that the differences in polymerase activity resulted from changes in the levels of these protein (Fig. 2).

## Discussion

The influenza A virus displays strong reassortment characteristics, which enable it to achieve adaptation in various species. In the present study, we demonstrated that the reassortant viruses with single pH1N1-origin segments (except for HA) in the backbone of the avian H9N2 influenza virus exhibited a comparable replication and transmission activity to the wild type H9N2 virus in turkeys and chickens. Additionally, introducing individual gene from avian H9N2 virus into pH1N1 virus allowed the reassortant viruses to acquire replication and/or transmission ability in avian species. These results revealed that once these pH1N1-avian reassortants generated, they could readily circulate in poultry.

It should be noted that some pH1N1-origin gene segments contribute to the transmissibility of the pH1N1 influenza virus, including PB2, NA, M, and NS genes^21^−^24^. Additionally, several pH1N1-H9N2 reassortant viruses possessed high pathogenicity and efficient respiratory droplet transmissibility in mammals^4^,^5^,^25^. Such reassortant viruses might further reassort with other avian influenza viruses in poultry. Therefore, the potential threat of pH1N1-H9N2 reassortant viruses for public health should be vigilance.

We found that the RNP activity of single PB2 gene reassortment were the lowest in both pH1N1 and H9N2 genetic background, and they resulted in the low replication and infectivity of the corresponding reassortants *in vitro* and *in vivo*. The data revealed that the PB2 genes of pH1N1 and H9N2 influenza viruses were incompatible with each other’s remaining genes. In contrast, the NA genes of BJ09 and HB08 were compatible to the genes from the other genetic background, which was characterized by the growth in CEF and replication and transmission ability in turkeys and chickens of BJ09-NA:HB08 and HB08-NA:BJ09 viruses were the best among the corresponding single-gene reassortants. Introducing the human-origin (including pH1N1) PA segment into the avian influenza virus could increase the polymerase activity and the adaptation of the avian virus in mammals^4^,^26^,^27^. It is interesting that the pH1N1-origin PA also enhanced the polymerase activity of the avian H9N2 virus in DF1 cells in the present study, indicating that the host origin of the PA gene was not related to the host range of the influenza virus. It is known that the PA gene of the pH1N1 virus was of North American avian origin, which might be compatible with the other gene segments of the H9N2 avian influenza virus^6^.

Turkeys are susceptible to a wide variety of influenza A viruses, including those from wild birds, swine and humans^15^,^28^,^29^. Therefore, turkeys are also a potential “mixing vessel” for influenza viruses. Outbreaks of pH1N1 infections in turkey breeder flocks were reported in several countries. However, although virus shedding and/or seroconversion could be detected in the experimentally inoculated turkeys, transmission between turkeys was not observed^29^,^30^,^31^,^32^. Different genetic characteristics between strains isolated from turkeys and humans might contribute to this inconsistency, and there are still no infection experiments in turkeys using pH1N1 clinically isolated from turkeys. Additionally, co-infection of other pathogens such as bacteria could reduce the immunity of turkeys resulting in turkeys that are more susceptible to pH1N1 infection. A recent study found that the pH1N1 virus was able to replicate and induce decreased egg production after experimental intrauterine inoculation^33^. These results revealed that pH1N1 might use other routes to achieve infection in turkeys. Additionally, other unknown predisposing factors expressed in the farm environment might transform turkeys into reservoirs of pH1N1 virus. Therefore, the possibility of reassortment between pH1N1 and avian influenza viruses in turkeys could not be excluded.

Chickens are poorly susceptible to pH1N1 virus; therefore they may not be “mixing vessel” for pH1N1 and avian influenza viruses. However, once the pH1N1-avian reassortant viruses emerged and prevailed, chickens could infect the viruses by contacting infected animals and humans or contaminated circumstance. Our results indicate that multiple pH1N1-avian reassortant viruses were able to replicate in chickens or even transmit between chickens. Chickens are the major terrestrial host for a wide variety of influenza viruses. Further reassortment of pH1N1-avian reassortants with other influenza viruses in chickens could not be excluded, which would make an epidemic situation of novel influenza viruses more complex and grim. Therefore, consistent surveillance of the influenza virus in poultry, avoidance of mixing the culture of livestock and poultry, and shutting down live poultry markets are important for controlling the novel influenza viruses at human-animal interface.

## Methods

### Ethics statement

All animal work was approved by the Beijing Association for Science and Technology (approval ID SYXK [Beijing] 2007-0023) and conducted in strict accordance with the Beijing Laboratory Animal Welfare and Ethics guidelines, as issued by the Beijing Administration Committee of Laboratory Animals, and in accordance with the China Agricultural University (CAU) Institutional Animal Care and Use Committee guidelines (ID: SKLAB-B-2011-003). The animal use protocol was approved by the Animal Welfare Committee of the CAU.

### Viruses and cells

pH1N1 virus A/Beijing/16/2009 (BJ09) and H9N2 virus A/chicken/Hebei/LC/2008 (HB08) were described previously, and the reverse genetics systems of these two viruses had been established^4^. CEF were derived from specific-pathogen-free chicken eggs. Briefly, after embryonating for 11 days, eggs were chilled at 4 °C and then disinfected by spraying their surface with 70% ethanol in water. Embryo fibroblasts were prepared according to standard methods^34^. CEF, human embryonic kidney cells (293T) and MDCK cells were grown in DMEM (Invitrogen) containing 10% fetal bovine serum (Invitrogen).

All experiments with live viruses were performed in a biosafety level 3 containment laboratory approved by the Ministry of Agriculture of the Peoples’s Republic of China.

### Generation of reassortant viruses by reverse genetics

A set of 16 “7 + 1” influenza viruses were generated by reverse genetics which included eight reassortants on the HB08 background replaced individually with a corresponding gene segment from BJ09 (denoted as BJ09-PB2:HB08, BJ09-PB1:HB08, BJ09-PA:HB08, BJ09-HA:HB08, BJ09-NP:HB08, BJ09-NA:HB08, BJ09-M:HB08, and BJ09-NS:HB08) and eight reassortants on the BJ09 background replaced individually with corresponding gene segment from HB08 (denoted as HB08-PB2:BJ09, HB08-PB1:BJ09, HB08-PA:BJ09, HB08-HA:BJ09, HB08-NP:BJ09, HB08-NA:BJ09, HB08-M:BJ09, and HB08-NS:BJ09) (Table 1).

Briefly, reverse transcription-PCR (RT-PCR) amplicons of the eight viral genes from HB08 and BJ09 viruses were cloned into a dual-promoter plasmid, PHW2000. MDCK and 293T cells were cocultured and transfected with 0.5 μg of each of the eight plasmids with corresponding constellation and 10 μL lipofectamine 2000 (Invitrogen) in a total volume of 1 mL of Opti-MEM (Invitrogen). After incubation at 37 °C for 6 h, the transfection mixture was removed from the cells and 2 mL of Opti-MEM containing 1 μg/mL of TPCK-trypsin was added. After 72 h, the supernatant was inoculated in 10-day-old SPF embryonated chicken eggs to produce stock viruses. Viral RNA was extracted and analyzed by RT-PCR, and each viral segment was sequenced to confirm the identity of the virus.

### Virus titrations and concentration

The 50% tissue culture infectious dose (TCID_50_) was determined in MDCK cells with 10-fold serially diluted viruses inoculated at 37 °C for 72 h. The 50% egg infectious dose (EID_50_) was determined in 10-day-old embryonated chicken eggs with 10-fold serially diluted viruses inoculated at 35 °C for 48 h. The TCID_50_ and EID_50_ values were calculated by the Reed-Muench method^35^.

Virus stocks were concentrated by ultracentrifugation. Briefly, stocks were clarified by low speed centrifugation (1250 g, 10 min, 4 °C), and centrifuged at 4 °C for 30 min at 18000 g; the supernatant was layered on an 10 ml cushion of 30% sucrose in Beckman Polyallomer Centriguge tubes (25 × 89 mm) and centrifuged in an SW28 rotor (Beckman) at 112000 g for 2.5 h at 4 °C; pellets were resuspended in PBS.

### Viral replication kinetics in CEF cells

Multistep replication kinetics were determined by inoculating CEF cells with a multiplicity of infection (MOI) of 0.001 TCID_50_ per cell in the presence of 0.5 μg/mL TPCK trypsin. Supernatants were sampled at 12, 24, 36, and 48 hpi. Virus titers in CEF cells were determined by TCID_50_. Three independent experiments were performed.

### Viral pathogenicity and transmission in turkeys and chickens

Six-week-old turkeys negative for influenza virus (n = 285) and SPF white leghorn chickens (n = 380) were randomly selected. Five turkeys and five chickens were inoculated intranasally with 10^6^ EID_50_ of each virus, except for HB08-PB2:BJ09 virus, in a 200-μL volume. To study viral transmission, the inoculated turkeys were placed in a new cage with five naïve turkeys and five naïve chickens, and the inoculated chickens were placed in a new cage with five naïve chickens. Oropharyngeal swabs were collected at 3 and 5 dpi [2 and 4 days post-contact (dpc)] for virus titration in embryonated chicken eggs. Three inoculated chickens in each virus group were euthanized at 3 dpi and lungs were collected for virus titration. Sera of the remaining birds were collected at 21 dpi (20 dpc) for seroconversion confirmation.

### Hemagglutination inhibition (HI) assay

The serum samples of turkeys were treated with receptor destroying enzyme (RDE; Denka Seiken, Ltd., Tokyo, Japan) (1 part serum; 3 parts RDE) for 18 h at 37 °C, followed by heat inactivation at 56 °C for 30 min. The HI assay was performed as previously described^36^. Briefly, 25 μL of serial twofold dilutions of the treated serum samples were mixed with 4 HA units of virus in V-shaped microtiter plates and incubated at room temperature for 30 minutes. Then, 25 μL of 1% chicken RBCs was added to each well and incubated at room temperature for 30 minutes. HI titers are regarded as being positive if there was inhibition at a serum dilution of 1 : 16^37^.

### RNP mini-genome luciferase assay

Four protein expression plasmids of PB2, PB1, PA and NP (50ng each) from either HB08 or BJ09 viruses was co-transfected into 293T cells together with the luciferase reporter plasmid p-Luci (10 ng) and internal control plasmid Renilla (5 ng). The assay was performed at both 37 °C and 39 °C. At 24 h post-transfection, cell lysate was prepared with Dual-Luciferase Reporter Assay System (Promega) and luciferase activity was measured using GloMax 96 microplate luminometer (Promega). PB2, PB1, PA, and NP expressions were confirmed by Western blotting. Primary antibodies were specific for influenza A virus PB2 (diluted 1 : 1000, GenScript, China), PB1 (diluted 1 : 3000, Thermo Fisher Scientific, USA), PA (1 : 3000, GeneTex, USA), and NP (diluted 1 : 3000, GeneTex, USA), respectively. The secondary antibody was horseradish peroxidase (HRP) - conjugated anti-rabbit antibody (diluted 1 : 10000 Jackson ImmunoResearch USA). HRP presence was detected using a western lightning chemiluminescence kit (Amersham Pharmacia, Freiburg, Germany), following the manufacturer’s protocol.

### Statistical analyses

Statistically significant differences between experimental groups were determined using the analysis of variance (ANOVA) method with the GraphPad Prism software package (GraphPad Software Inc., La Jolla, CA). A *P* value <0.05 was considered statistically significant.

## Additional Information

**How to cite this article**: Sun, H. *et al*. The infection of turkeys and chickens by reassortants derived from pandemic H1N1 2009 and avian H9N2 influenza viruses. *Sci. Rep.* **5**, 10130; doi: 10.1038/srep10130 (2015).

## Figures and Tables

**Table 1:**
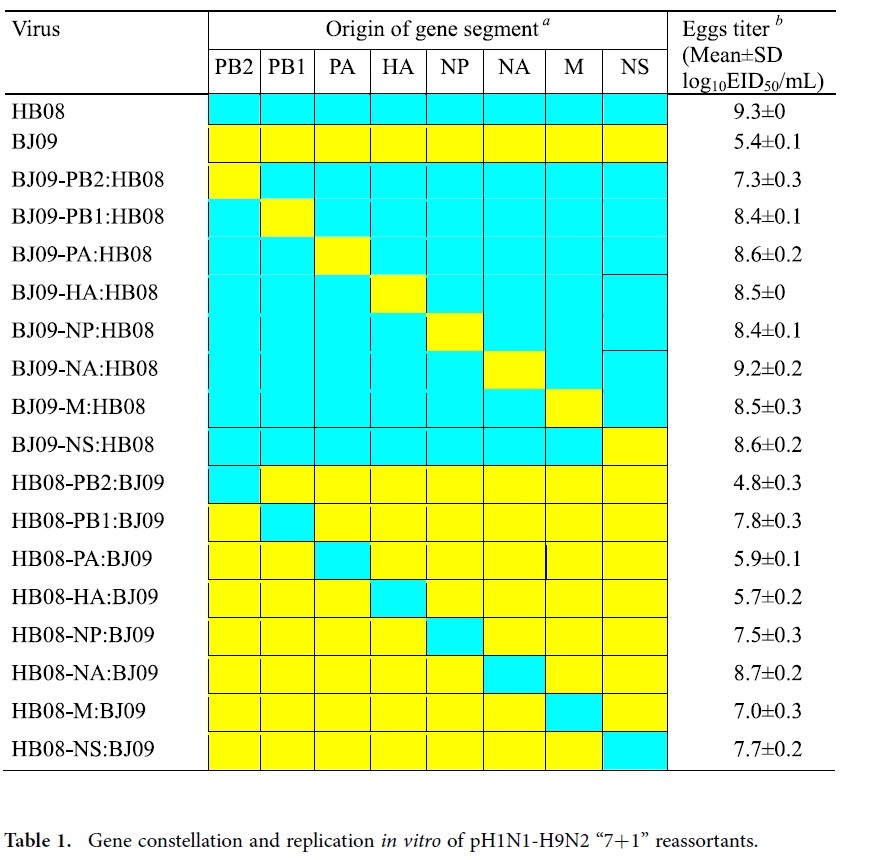
Gene constellation and replication *in vitro* of pH1N1-H9N2 “7+1” reassortants.

**Table 2:** Replication and transmission in turkeys and turkey-to-chicken transmission of pH1N1-H9N2 “7+1” reassortants.

Virus	Inoculated turkeys			Contact turkeys			Contact chickens		
	3dpi	5dpi	Seroconversion	2dpc	4dpc	Seroconversion	2dpc	4dpc	Seroconversion
HB08	10/10 (4.3 ± 0.4)^a^	10/10 (4.1 ± 0.8)	10/10 (512–1024)^b^	4/5 (2.2 ± 0.5)	5/5 (3.9 ± 0.5)	5/5 (128–1024)	2/5 (2.5 ± 0.2)	4/5 (3.4 ± 0.3)	5/5 (512–1024)
BJ09	0/10	0/10	3/10 (64)	0/5	0/5	0/5	0/5	0/5	0/5
BJ09-PB2:HB08	9/10 (3.7 ± 0.7)	3/10 (2.8 ± 0.3)	10/10 (256–1024)	0/5	1/5 (1.8)	1/5 (256)	2/5 (2.4 ± 0.2)	2/5 (2.5 ± 0.2)	2/5 (256)
BJ09-PB1:HB08	10/10 (3.9 ± 0.8)	5/10 (3.2 ± 0.3)	10/10 (256–1024)	1/5 (2.3)	2/5 (2.1 ± 0.3)	2/5 (32–512)	2/5 (3.2 ± 0.3)	3/5 (3.9 ± 0.5)	5/5 (256–1024)
BJ09-PA:HB08	10/10 (4.0 ± 0.5)	6/10 (3.5 ± 0.4)	10/10 (512–1024)	1/5 (1.8)	1/5 (1.8)	2/5 (64–128)	1/5 (2.5)	2/5 (2.8 ± 0.4)	3/5 (256–512)
BJ09-HA:HB08	7/10 (2.5 ± 0.6)	0/10	8/10 (512–1024)	0/5	0/5	1/5 (64)	0/5	1/5 (1.8)	2/5 (32–64)
BJ09-NP:HB08	10/10 (4.0 ± 0.8)	7/10 (3.5 ± 0.3)	10/10 (512–1024)	1/5 (2.3)	1/5 (1.8)	2/5 (128–256)	2/5 (2.9 ± 0.2)	2/5 (3.0 ± 0.3)	5/5 (256–512)
BJ09-NA:HB08	10/10 (4.2 ± 1.1)	5/10 (3.3 ± 0.4)	10/10 (256–1024)	5/5 (2.9 ± 0.3)	5/5 (4.1 ± 0.3)	5/5 (128–256)	5/5 (3.8 ± 0.3)	4/5 (2.9 ± 0.5)	5/5 (1024–2048)
BJ09-M:HB08	10/10 (3.9 ± 0.8)	4/10 (3.1 ± 0.3)	10/10 (512–1024)	5/5 (2.5 ± 0.4)	5/5 (3.6 ± 0.3)	5/5 (256–512)	5/5 (3.5 ± 0.2)	5/5 (2.5 ± 0.7)	5/5 (512–2048)
BJ09-NS:HB08	10/10 (3.8 ± 0.6)	4/10 (3.2 ± 0.5)	10/10 (128–1024)	5/5 (2.6 ± 0.3)	5/5 (3.8 ± 0.4)	5/5 (128–256)	5/5 (3.8 ± 0.8)	4/5 (2.7 ± 0.3)	5/5 (128–1024)
HB08-PB2:BJ09	ND^c^	ND	ND	ND	ND	ND	ND	ND	ND
HB08-PB1:BJ09	4/10 (2.3 ± 0.4)	5/10 (2.9 ± 0.3)	10/10 (128–512)	1/5 (1.8)	3/5 (2.0 ± 0.5)	4/5 (128–512)	0/5	1/5 (1.8)	2/5 (64–256)
HB08-PA:BJ09	2/10 (1.8 ± 0)	3/10 (2.1 ± 0.4)	8/10 (64–512)	1/5 (1.8)	3/5 (1.9 ± 0.4)	4/5 (256–512)	0/5	0/5	3/5 (256–512)
HB08-HA:BJ09	3/10 (1.8 ± 0)	6/10 (2.5 ± 0.4)	10/10 (256–512)	0/5	0/5	0/5	0/5	0/5	0/5
HB08-NP:BJ09	2/10 (2.1 ± 0.2)	7/10 (2.8 ± 0.5)	10/10 (64–512)	0/5	3/5 (2.2 ± 0.3)	4/5 (256–512)	0/5	0/5	2/5 (256)
HB08-NA:BJ09	1/10 (1.8)	5/10 (2.8 ± 0.3)	9/10 (64–512)	1/5 (1.8)	3/5 (2.3 ± 0.2)	5/5 (128–512)	0/5	2/5 (1.8 ± 0)	2/5 (128–512)
HB08-M:BJ09	0/10	0/10	4/10 (32–128)	0/5	0/5	0/5	0/5	0/5	0/5
HB08-NS:BJ09	2/10 (1.8 ± 0)	4/10 (2.4 ± 0.5)	8/10 (256–512)	2/5 (1.8)	3/5 (2.3 ± 0.3)	5/5 (256–512)	0/5	2/5 (1.8 ± 0)	3/5 (128–512)

^a^Number of positive oropharyngeal swabs/total number (mean titers [log_10_ EID_50_/mL]  ±  SD). Serum was collected on 21 dpi, and BJ09 or HB08 viruses were used in HI assays.

^b^Number of positive birds/total number (rang of HI titers)

^c^ND, not done.

**Table 3:** Replication and transmission in chickens of pH1N1-H9N2 “7+1” reassortants.

**Virus**	**Inoculated chickens**				**Contact chickens**		
	**3dpi**	**5dpi**	**lung**	**Seroconversion**	**2dpc**	**4dpc**	**Seroconversion**
HB08	10/10 (4.6 ± 0.8)^a^	6/7 (3.9 ± 0.2)	3/3 (4.9 ± 0.3)	7/7 (1024–2048)^b^	2/5 (3.7 ± 0.2)	5/5 (3.8 ± 0.3)	5/5 (256–1028)
BJ09	0/10	0/7	0/3	0/7	0/5	0/5	0/5
BJ09-PB2:HB08	9/10 (3.0 ± 0.4)	2/7 (2.5 ± 0.1)	1/3 (3.3)	7/7 (512–2048)	0/5	1/5 (1.8)	2/5 (256–1024)
BJ09-PB1:HB08	10/10 (4.3 ± 0.7)	3/7 (3.6 ± 0.1)	3/3 (5.0 ± 1.8)	7/7 (512–1024)	3/5 (3.5 ± 0.5)	3/5 (3.8 ± 0.4)	5/5 (256–1280)
BJ09-PA:HB08	9/10 (4.0 ± 0.6)	2/7 (3.8 ± 0)	2/3 (4.4 ± 0.2)	7/7 (1024–2048)	1/5 (1.8)	3/5 (2.4 ± 0.4)	4/5 (128–1024)
BJ09-HA:HB08	1/10 (1.8)	0/7	1/3 (1.8)	6/7 (1024–2048)	0/5	1/5 (1.8)	2/5 (256-512)
BJ09-NP:HB08	9/10 (4.0 ± 0.5)	2/7 (3.6 ± 0.3)	2/3 (4.5 ± 1.8)	7/7 (512-1024)	1/5 (1.8)	3/5 (1.8 ± 0)	3/5 (128-512)
BJ09-NA:HB08	10/10 (5.0 ± 1.1)	7/7 (4.0 ± 0.6)	3/3 (4.9 ± 0.8)	7/7 (1024-2048)	4/5 (3.7 ± 0.2)	5/5 (4.0 ± 0.4)	5/5 (256-2048)
BJ09-M:HB08	9/10 (4.6 ± 0.8)	5/7 (4.1 ± 0.8)	3/3 (4.8 ± 1.5)	7/7 (1024-2048)	3/5 (2.7 ± 0.2)	5/5 (3.6 ± 0.1)	5/5 (128-2048)
BJ09-NS:HB08	8/10 (4.3 ± 0.7)	5/7 (3.6 ± 0.3)	3/3 (4.0 ± 1.1)	7/7 (1024-2048)	3/5 (2.8 ± 0.3)	5/5 (3.5 ± 0.1)	5/5 (256–2048)
HB08-PB2:BJ09	ND^c^	ND	ND	ND	ND	ND	ND
HB08-PB1:BJ09	3/10 (2.5 ± 0.3)	0/7	0/3	7/7 (512–2048)	0/5	2/5 (1.9 ± 0.1)	3/5 (256–1024)
HB08-PA:BJ09	2/10 (2.1 ± 0.4)	0/7	1/3 (1.8)	7/7 (512–1024)	0/5	0/5	2/5 (128–512)
HB08-HA:BJ09	3/10 (1.8 ± 0)	0/7	0/3	7/7 (256–1024)	0/5	0/5	0/5
HB08-NP:BJ09	4/10 (2.3 ± 0.3)	0/7	0/3	7/7 (1024)	0/5	1/5 (1.8)	3/5 (256–1024)
HB08-NA:BJ09	4/10 (2.7 ± 0.3)	0/7	0/3	7/7 (512–1024)	0/5	2/5 (1.9 ± 0.1)	5/5 (128–512)
HB08-M:BJ09	0/10	0/7	0/3	2/7 (128–512)	0/5	0/5	0/5
HB08-NS:BJ09	3/10 (2.6 ± 0.4)	0/7	0/3	7/7 (1024–2048)	1/5 (1.8)	2/5 (1.9 ± 0.1)	5/5 (512–1024)

^a^Number of positive oropharyngeal swabs/total number (mean titers [log_10_ EID_50_/mL] ± SD). Serum was collected on 21 dpi, and BJ09 or HB08 viruses were used in HI assays.

^b^Number of positive chickens/total number (rang of HI titers)

^c^ND, not done.
